# Investigating the Prognostic Value of Pretreatment Body Composition in Women with Ovarian Cancer: Impact on Clinical Outcomes

**DOI:** 10.3390/cancers18091478

**Published:** 2026-05-04

**Authors:** Sarah Benna-Doyle, Erin Laing, Brenton J. Baguley, Nicholas Hardcastle, Gavin Abbott, Nicole Kiss

**Affiliations:** 1 Institute for Physical Activity and Nutrition, Deakin University, Geelong, VIC 3216, Australia; nicole.kiss@deakin.edu.au (N.K.); 2 School of Exercise and Nutrition Sciences, Deakin University, Burwood, VIC 3125, Australia; 3 Nutrition and Speech Pathology Department, Peter MacCallum Cancer Centre, Melbourne, VIC 3000, Australia; 4 Sir Peter MacCallum Department of Oncology, University of Melbourne, Melbourne, VIC 3000, Australia; 5 Department of Radiation Oncology, Peter MacCallum Cancer Centre, Melbourne, VIC 3000, Australia

**Keywords:** ovarian cancer, body composition, survival, muscle mass, menopause, treatment outcomes

## Abstract

Background/Objectives: In ovarian cancer, the association between low muscle mass and outcomes is unclear, despite well-established evidence in other cancer diagnoses. Overall body composition may have greater prognostic value. This study aimed to investigate associations between body composition and clinical outcomes. Methods: Retrospective study of women diagnosed with ovarian cancer between August 2020 and March 2024. Clinical characteristics were extracted from medical records. Body composition phenotypes (low skeletal muscle index [SMI], low skeletal muscle density [SMD], high subcutaneous [SATI], visceral [VATI] and total adipose tissue index [TATI]) were assessed from pre-treatment CT images. Logistic regression, chi-squared, Fisher’s exact tests, and Mann–Whitney U tests were used to assess associations between body composition phenotypes, patient characteristics and study outcomes. Cox proportional hazards models were fitted to examine associations with survival. Results: Ninety-nine patients [mean (SD) age 61 (12.7) years, 80% stage III/IV] were included. Overall, 67 (67%) women had low SMI, 57 (57%) low SMD, and 49 (49%) high TATI. Thirty-seven (37%) women had low SMI and low SMD, 23 (23%) low SMI with high TATI, and 33 (33%) low SMD with high TATI. Post-menopausal status was associated with higher VAT (*p* = 0.004). Low SMI or low SMD co-occurring with high adiposity was associated with a 1.91–2.03 and 2.15–2.49 higher hazard mortality. There was insufficient or weak evidence of an association between body composition and other treatment outcomes. Conclusion: These findings suggest additive effects when phenotypes co-occur. In ovarian cancer, singular assessments may be an oversimplification. Future studies should continue to consider co-occurring phenotypes to adequately capture risk.

## Simple Summary

Ovarian cancer has the poorest survival outcomes among all gynaecological cancers. Body composition has been identified as a modifiable patient-related factor with the potential to enhance how patients are evaluated to identify risk of poorer outcomes. Our retrospective study aimed to identify pretreatment body composition phenotypes in women with ovarian cancer and investigate relationships with treatment outcomes, including survival. In this cohort of 99 women, we found most women had low muscle mass, over half had poor muscle composition, and only four had a normal body composition before starting treatment. We also found that post-menopausal women had higher amounts of visceral fat. Women who had low muscle mass or poor muscle composition occurring alongside high adiposity before treatment had the poorest survival outcomes. These findings suggest potentially additive effects when body composition phenotypes occur together. Future studies should continue to consider the complexity of body composition when assessing pretreatment risk.

## 1. Introduction

Globally, cancer is a leading cause of death [1]. The increasing pressure on health systems in every country, as the burden of cancer continues to grow, requires high-quality, equitable care across cancer diagnoses [1]. Ovarian cancer continues to have the poorest survival outcomes among all gynaecological cancers worldwide [2]. In Australia, ovarian cancer accounts for 26% of all gynaecological cancers, yet is responsible for 48% of all gynaecological cancer deaths [3]. Most women are diagnosed with an advanced-stage, high-grade disease, and less than 50% will survive five years [4]. Reducing these discrepancies requires care equipped to address the entire cancer continuum, including innovations in predictive biomarkers to tailor treatment and enhance risk stratification [1,5].

Maximal cytoreductive surgery with platinum-based chemotherapy prior to (neoadjuvant) and/or after (adjuvant) surgery remain central in the treatment of ovarian cancer [6,7,8]. The ability to tolerate, adhere to and recover from the prescribed treatment schedule, including the achievement of maximal cytoreduction, is highly dependent upon both tumour- and patient-related factors [9]. While established clinical prognostic factors such as age at diagnosis, disease stage, histological subtype, performance status, menopausal status, ascites and degree of residual disease following surgery are well-established in ovarian cancer [10,11], increasing attention is being placed upon modifiable patient-related factors. Among them, computed tomography (CT) quantification of muscle morphology (mass and composition) and body composition, specifically the distribution of muscle and adipose tissue, have been identified as modifiable factors with predictive and prognostic significance [12,13].

In other cancer diagnoses, low muscle mass is consistently associated with increased postoperative complications, treatment-related toxicity, and overall survival [14,15,16]; however, these associations in ovarian cancer are not as clear. Several meta-analyses have investigated the prognostic value of low muscle mass in ovarian cancer with mixed results [11,17,18,19,20,21,22]. Similarly, the relationship between overweight and obesity and survival outcomes in ovarian cancer is unclear [23,24]. This uncertainty may in part be explained by the limitations of using body mass index (BMI) as a proxy for adiposity [25,26]. More recently, a meta-analysis of CT assessed adiposity by Cheng et al. [27] reported no associations between visceral, subcutaneous or total adiposity and survival outcomes in ovarian cancer, with data pooled from four studies. Low muscle mass and adiposity, however, represent single body composition phenotypes that are not mutually exclusive [28]. The cumulative burden of low muscle mass co-existing with high adiposity and low muscle quality may have greater prognostic significance [17,18,20,29,30,31,32,33]. Given the majority of women with ovarian cancer (~75%) are diagnosed at a menopausal age, this population also represents an important intersection between cancer and age-related changes in body composition [34]. The composition of the skeletal muscle, which reflects the degree of lipid deposition into muscle, is represented by skeletal muscle radiodensity (SMD), a prognostic marker inversely related to muscle lipid deposition [35]. Across the ovarian cancer literature, low SMD is consistently associated with poorer overall survival [17,18,20,29,30,31] and may be a more robust predictor for postoperative complications [20].

To date, most research in ovarian cancer has evaluated a singular muscle phenotype, primarily low muscle mass, and focused on survival outcomes, postoperative complications and hospitalisation [11,17,21]. More recently, investigations have begun to examine the differential and combined effects of adipose tissue compartments with muscle mass on survival, and chemotoxicity [29,30,31,32]. However, across all investigations, co-occurring low SMD has not yet been considered. Earlier research in other cancer types has shown that while low muscle mass and low SMD are distinct phenotypes, when they occur together, they have additive effects [36,37]. In patients with pancreatic and periampullary cancers (*n* = 123), neither low skeletal muscle index (SMI) nor SMD alone was associated with worse overall survival, whereas the co-occurring SMI and SMD phenotype was associated with 3.08-fold increased hazard for mortality (95% CI: 1.01–9.43, *p* = 0.048) [36], and similar associations were observed in colorectal cancer [37]. Given the multifaceted nature of body composition, comprehensive assessment that considers the differential and combined effects of all potential phenotype combinations, including low SMD, is required to build on the emerging body composition research.

Accordingly, this study comprehensively evaluates pretreatment body composition and its associations with key treatment-related outcomes in a cohort of women diagnosed with epithelial ovarian cancer. First, muscle mass, SMD, and adipose tissue areas (visceral, subcutaneous, total) were quantified from secondary analysis of routine CT imaging. We then examined pretreatment body composition phenotypes and associations between independently and concurrently occurring phenotypes with surgical outcomes, chemotherapy-related outcomes, hospitalisation and survival. This approach considers the influences of all body composition phenotypic combinations, extending the literature beyond singular assessments across several clinically important outcomes, including chemotoxicity.

## 2. Materials and Methods

### 2.1. Ethics Approval and Reporting

This study was approved by the Peter MacCallum Cancer Centre Human Research Ethics Committee with waiver of informed consent (24/39L, HREC/1066859/PMCC) and Deakin University Research Ethics. This study is reported according to the Strengthening the Reporting of Observational studies in Epidemiology (STROBE) statement [38].

### 2.2. Study Design and Population

A retrospective, observational cohort study was conducted in patients newly diagnosed with epithelial ovarian cancer (EOC) who received first-line treatment at Peter MacCallum Cancer Centre from the inception of electronic medical records (EMRs) in September 2020 until March 2024. Inclusion criteria included adult females (≥18 years), a histologically confirmed diagnosis of EOC, and the availability of any pretreatment CT image acquired for staging, diagnosis, or treatment planning. Exclusion criteria included a previous or concurrent cancer diagnosis, co-morbidities known to affect body composition (e.g., muscular dystrophy, other degenerative muscular conditions), CT images without suitable quality for analysis or images that did not contain the third lumbar vertebrae (L3).

### 2.3. Clinical Data Extraction

Baseline patient demographics, clinicopathological data, ovarian cancer-specific characteristics and treatment schedules were extracted from the EMR. Demographic and clinicopathological data included age at diagnosis, weight (kg), height (cm), BMI (kg/m^2^), Charlson co-morbidity score [39], Eastern Cooperative Oncology group (ECOG) performance status [40], and menopausal status (postmenopausal, yes/no). Ovarian cancer-specific data included International Federation of Gynaecology and Obstetrics stage of disease (stage I to IV) [41] and histological subtype(s) (high-grade serous, low-grade serous, endometrioid, clear cell, mucinous, other). Treatment schedules for chemotherapy (neoadjuvant and/or adjuvant), surgery type, including primary debulking (PDS), interval debulking surgery (IDS), and surgery with intraperitoneal chemotherapy (IP) (hyperthermic or normothermic), and any additional treatment with radiotherapy, hormone therapy, and targeted therapies (bevacizumab and PARP inhibitors) were extracted from the EMR.

### 2.4. Body Composition Analysis by Computed Tomography

Routine pretreatment CT images acquired for staging and treatment planning were assessed. SliceOmatic^®^ software Version 6.0 (Tomovision, Quebec, QC, Canada) was used to assess body composition and quantify skeletal muscle area (SMA), SMD, and adipose tissue areas (visceral and subcutaneous) using the Alberta Protocol [42,43]. A range of −29 to 150 Hounsfield Units (HU) was used to distinguish SMA at L3. SMA was normalised for height (m^2^) to determine SMI (cm^2^/m^2^). For SMD (representing muscle composition), the mean muscle attenuation (HU) was reported. Low SMI was defined as <41 cm^2^/m^2^ for patients of any BMI. Low SMD was defined by a threshold of <41 HU for patients with a BMI ≤ 24.5 kg/m^2^ and <33 HU for patients with a BMI ≥ 25.0 kg/m^2^ [44]. The thresholds used to define low SMI and SMD were according to the widely used sex-and-BMI-specific cut-points proposed by Martin et al. [44], derived from a Canadian cancer population and appropriately similar to the Australian population in our study.

For adipose tissues, a range of −30 to −190 HU was used to distinguish subcutaneous adipose tissue (SAT), and a range of −50 to −150 HU to distinguish visceral adipose tissue (VAT). The sum of SAT and VAT were normalised for height (m^2^) to generate total adipose tissue index (TATI cm^2^/m^2^). The cross-sectional areas for SAT and VAT individually were normalised for height (m^2^) to generate SATI (cm^2^/m^2^) and VATI (cm^2^/m^2^). In the absence of validated cut-off points to define high adipose tissue areas, SATI, VATI and TATI were dichotomised using the median as the cut-point, a sample-derived approach used in other studies [45,46]. All segmentation was completed by a single certified researcher (SBD) and reviewed for accuracy by a second researcher with expertise in this methodology (NK).

### 2.5. Outcome Measures

Surgical outcomes, including the occurrence and severity of postoperative complications and degree of residual disease following surgery, were extracted from medical records. Postoperative complications were classified according to the Clavien–Dindo scale of surgical complications [47], with a complication grade of ≥3 used to define severe complications. Degree of residual disease following surgery was reported as complete (no macroscopic disease), optimal debulk (≤1 cm) or suboptimal debulk (>1 cm) [8].

Hospitalisation outcomes included length of surgical admission and 30-day readmissions following surgery. Readmission was defined as any inpatient or emergency admission within 30 days of first surgical discharge.

Chemotherapy-related outcomes included time (in days) to commencement of adjuvant chemotherapy, toxicities from the first chemotherapy cycle, dose modifications, reductions in number of planned cycles and discontinuation during any schedule (neo/adjuvant). Chemotherapy toxicities were defined using the Common Terminology Criteria for Adverse Events (CTCAE) [48], and when documented, the complication grade (Grade 0 [nil] to 4 [life threatening]) were extracted from the medical records.

Overall survival was defined as the date of diagnosis until death of any cause or date of censoring 2 July 2025.

Death data were ascertained from the Victorian Registry of Births, Deaths and Marriages. Death dates were reviewed and cross-referenced with the hospital medical records, which are immediately updated to account for reporting delays, deaths outside jurisdiction and limitations to data matching with registry data [49].

### 2.6. Statistical Analysis

Descriptive statistics were reported as mean ± SD or median [IQR] for continuous variables and counts (percentages) for categorical variables. Continuous variables were checked for normality using histograms and Q-Q plots. There was <5% missing data overall, and all analyses were conducted on an available case basis. Patient and clinical characteristics between body composition phenotypes (e.g., low SMI vs. normal SMI, low SMD vs. normal SMD, high TATI vs. normal TATI) were compared using chi-squared and Fisher’s exact tests for categorical variables and independent t-tests and Mann–Whitney U tests for continuous variables. The differential and combined effects (where sample size permitted) of each body composition phenotype on treatment-related outcomes were evaluated using Mann–Whitney U tests for continuous outcomes, chi-squared or Fisher’s exact tests for associations between categorical variables and logistic regression for binary outcomes. Associations between pretreatment body composition with complication grade, 30-day readmission, chemotherapy toxicity and dose modifications were examined using chi-squared and Fisher’s exact tests. Associations with LOS and time to adjuvant chemotherapy were assessed using Mann–Whitney U tests due to non-normality. Potential confounding factors were identified a priori using directed acyclic graphs (DAGs) with DAGitty software version 3.1 [50,51]. Age (continuous), tumour stage (binary) and CCI (continuous) were identified as potential confounders, with BMI (continuous) an additional potential confounder for low SMD models (Supplementary Material Figure S1). Logistic regression models were fitted to examine postoperative complication occurrence by phenotype. Given the limited number of events, the models were adjusted for age and tumour stage based on their strong prognostic associations with survival in ovarian cancer [51] and their influence on pretreatment body composition.

**Figure 1:**
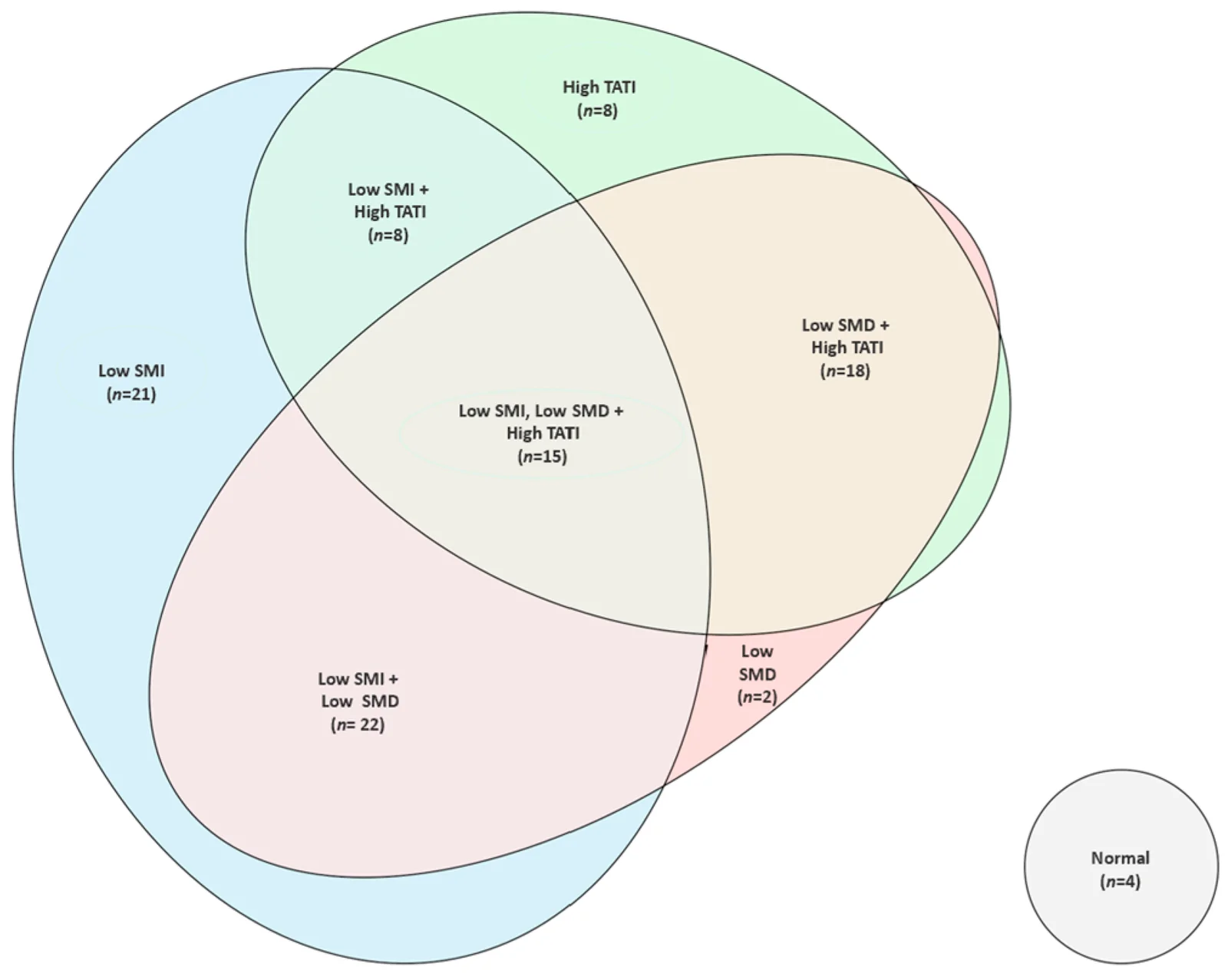
Euler diagram demonstrating proportion of each body composition phenotype across the sample.

Cox proportional hazards models were fitted and adjusted to estimate the association between pretreatment body composition phenotype and survival. All models were adjusted for age, tumour stage and CCI. Low SMD models were further adjusted for BMI. The proportional hazards assumptions were tested and met for all models. Leave-one-out analysis was performed, and no influential observations were identified. Covariate-adjusted Kaplan–Meier curves were constructed for overall survival for each body composition phenotype. All statistical analyses were two-sided and conducted in Stata/SE version 18.0 (College Station, TX USA). Figure 1 was generated using R version 4.4.0 (Vienna, Austria). The strength of the evidence against the null hypothesis (i.e., no association) was interpreted as: *p* < 0.001 very strong evidence, *p* < 0.01 strong evidence, *p* < 0.05 moderate evidence, *p* < 0.1 weak evidence, *p* > 0.1 insufficient evidence [52]. As analysis was considered exploratory, no adjustment was made for multiple testing.

## 3. Results

A total of 99 patients were included in the study. Patient characteristics are described in Table 1. Mean age at diagnosis was 61 ± 12.7 (range 28–89), 67% (*n* = 66) had high-grade serous histology, and 83% (*n* = 80) had an advanced stage of disease (III/IV). Of the included women, 75 (76%) were treated with surgery, 24 (24%) with neoadjuvant chemotherapy only, 19 (19%) with adjuvant chemotherapy only, 33 (33%) received both schedules, while 32 (32%) received maintenance targeted therapy. Overall, 67% had low SMI, 57% had low SMD and 37% had both low SMI and SMD prior to commencing treatment. Using the median (≥107.9 cm^2^/m^2^) to define high total adiposity, 49% had high TATI. Among them, 23% had high TATI with low SMI, 33% high TATI with low SMD and 15% had high TATI with low SMI and low SMD. When evaluating the distribution of each body composition phenotype exclusively, only four patients had normal body composition at treatment commencement (Figure 1). The mean time between CT scan and treatment commencement was 20.7 ± 24.7 days.

### 3.1. Associations Between Patient Characteristics and Body Composition

**Figure 2:**
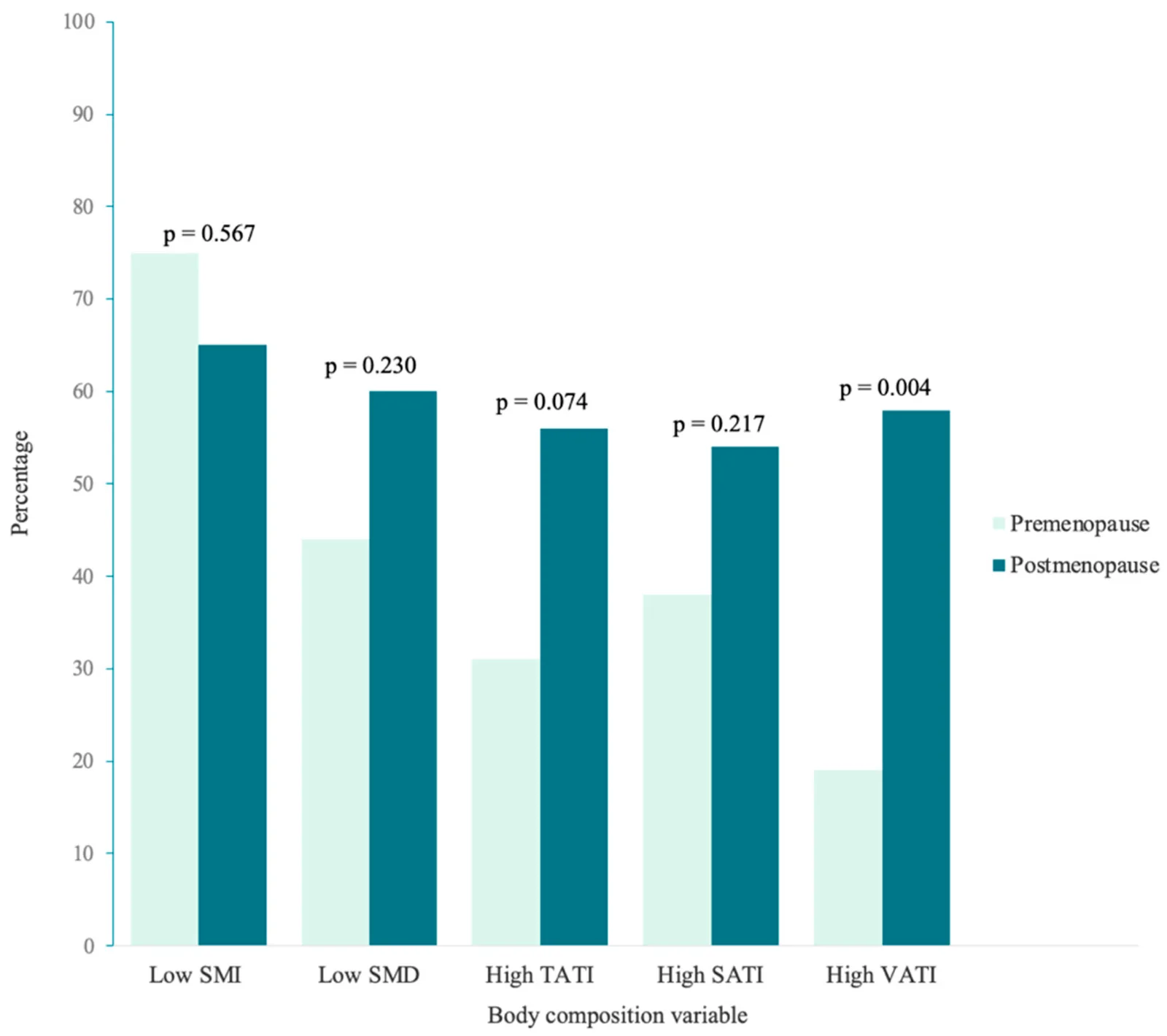
Prevalence of body composition phenotypes by menopausal status.

There was very strong evidence that both low body weight and low BMI were associated with low SMI (*p* < 0.001). For SMD, there was strong evidence that an impaired performance status was associated with low SMD (*p* = 0.006), moderate evidence that older age (*p* = 0.012), a higher BMI (*p* = 0.012) and higher body weight (*p* = 0.048) were associated with low SMD and weak evidence that a higher comorbidity score was associated with low SMD (*p* = 0.057). There was very strong evidence that a higher body weight (*p* < 0.001) and higher BMI (*p* < 0.001), moderate evidence that a higher comorbidity score (*p* = 0.017) and weak evidence that post-menopausal status (*p* = 0.074) were associated with high TATI. When investigating body composition by menopausal status, there was strong evidence of an association between post-menopausal status and high VATI (*p* = 0.004), weak evidence of an association with high TATI (*p* = 0.074), and no evidence of an association with high SATI (*p* = 0.217). There was no evidence of association between skeletal muscle phenotypes (low SMI and low SMD) and menopausal status. Body composition by menopausal status is shown in Figure 2. No other variables were associated with pretreatment body composition (Table 2).

### 3.2. Surgical Outcomes

Of the 75 patients treated with surgery, 34 (45%) experienced a postoperative complication. The most common complications were non-infections (79%, *n* = 27), with 35% (*n* = 12) requiring a blood transfusion, and gastrointestinal complications (35%, *n* = 12), with 16% (*n* = 9) experiencing postoperative ileus. Eighteen (53%) patients had multiple complications, five (15%) were admitted to intensive care following surgery, and two (6%) were returned to theatre due to complications. There was no evidence of an association between any body composition phenotype and postoperative complications (Supplementary Table S1). Median LOS for surgical admission was 3 [2-7] days, and 10 (13%) women were readmitted within 30 days of surgical discharge. There was no evidence of an association between any body composition phenotype with LOS, and weak evidence that high TATI was associated with fewer 30-day readmissions (*p* = 0.086) (Table 3). Of the 34 women who experienced a postoperative complication, 23 (68%) were minor, and 11 (32%) were severe (Clavien–Dindo classification ≥ grade 3). There was no evidence of an association between any body composition phenotype and complication severity (Table 3).

### 3.3. Chemotherapy-Related Outcomes

Of the 57 women treated with neoadjuvant chemotherapy, 42 (74%) experienced toxicity (≥Grade 1) from chemotherapy cycle one, and 27 (47%) required a dose modification. For those treated with adjuvant chemotherapy following surgery (*n* = 52), median time to chemotherapy commencement was 24.5 [21-29] days (range 2–63), 31 (60%) experienced a toxicity (≥Grade 1) from the first cycle, and 33 (63%) required a dose modification. There was no evidence of an association between neoadjuvant chemotherapy-related outcomes and any body composition phenotype. For adjuvant chemotherapy, there was weak evidence of an association between low SMI and low SMD combined phenotype and shorter time to adjuvant chemotherapy (*p* = 0.050). There was also weak evidence that high VATI was associated with reduced toxicity from adjuvant chemotherapy cycle one (*p* = 0.089) (Table 3).

### 3.4. Survival

**Figure 3:**
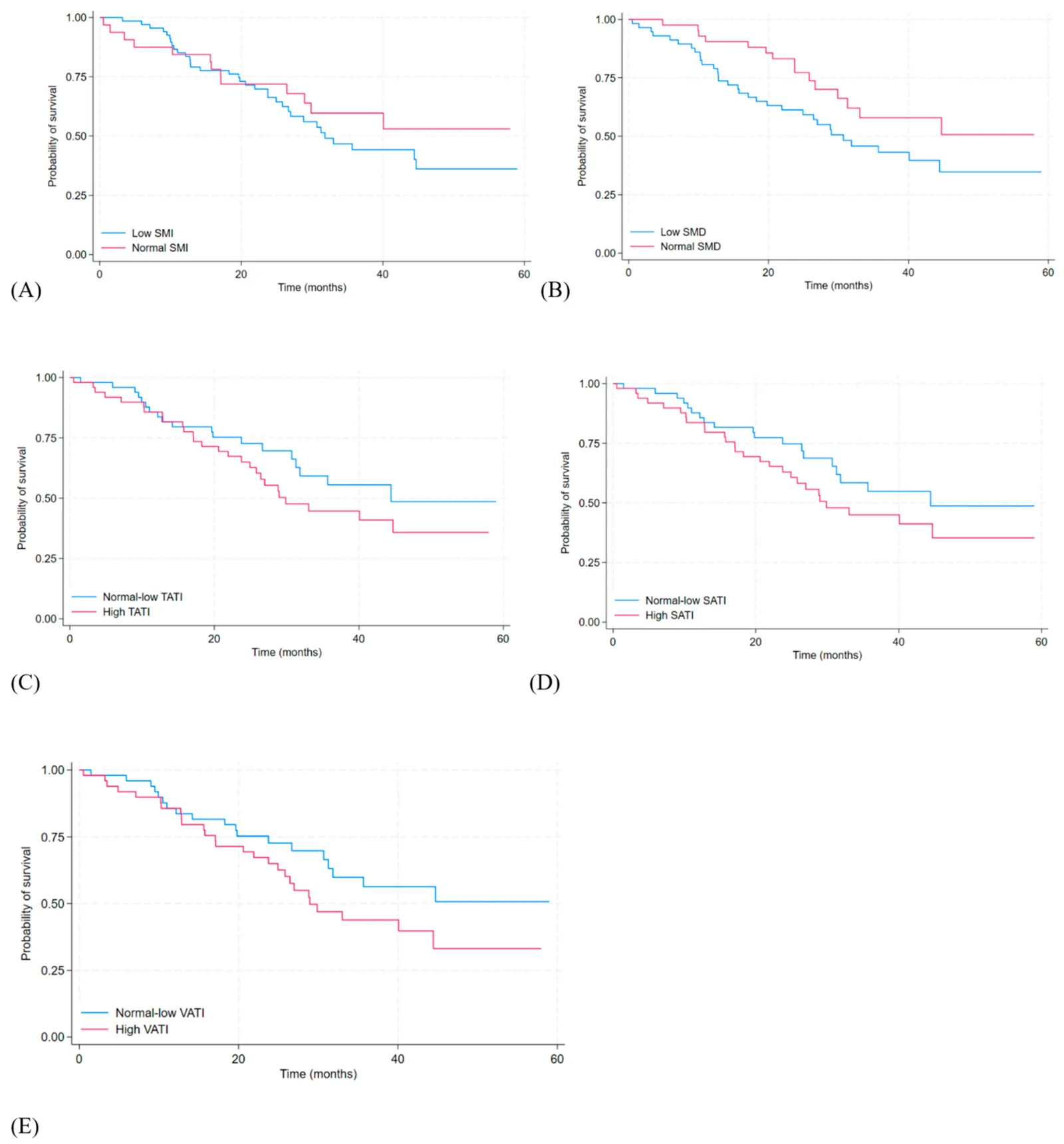
Kaplan–Meier survival curves for singular phenotypes: (**A**) low SMI, (**B**) low SMD, (**C**) high TATI, (**D**) high SATI, (**E**) high VATI. SMI: skeletal muscle index, SMD: skeletal muscle density, TATI: total adipose tissue, SATI: subcutaneous adipose tissue index, VATI: visceral adipose tissue index.

**Figure 4:**
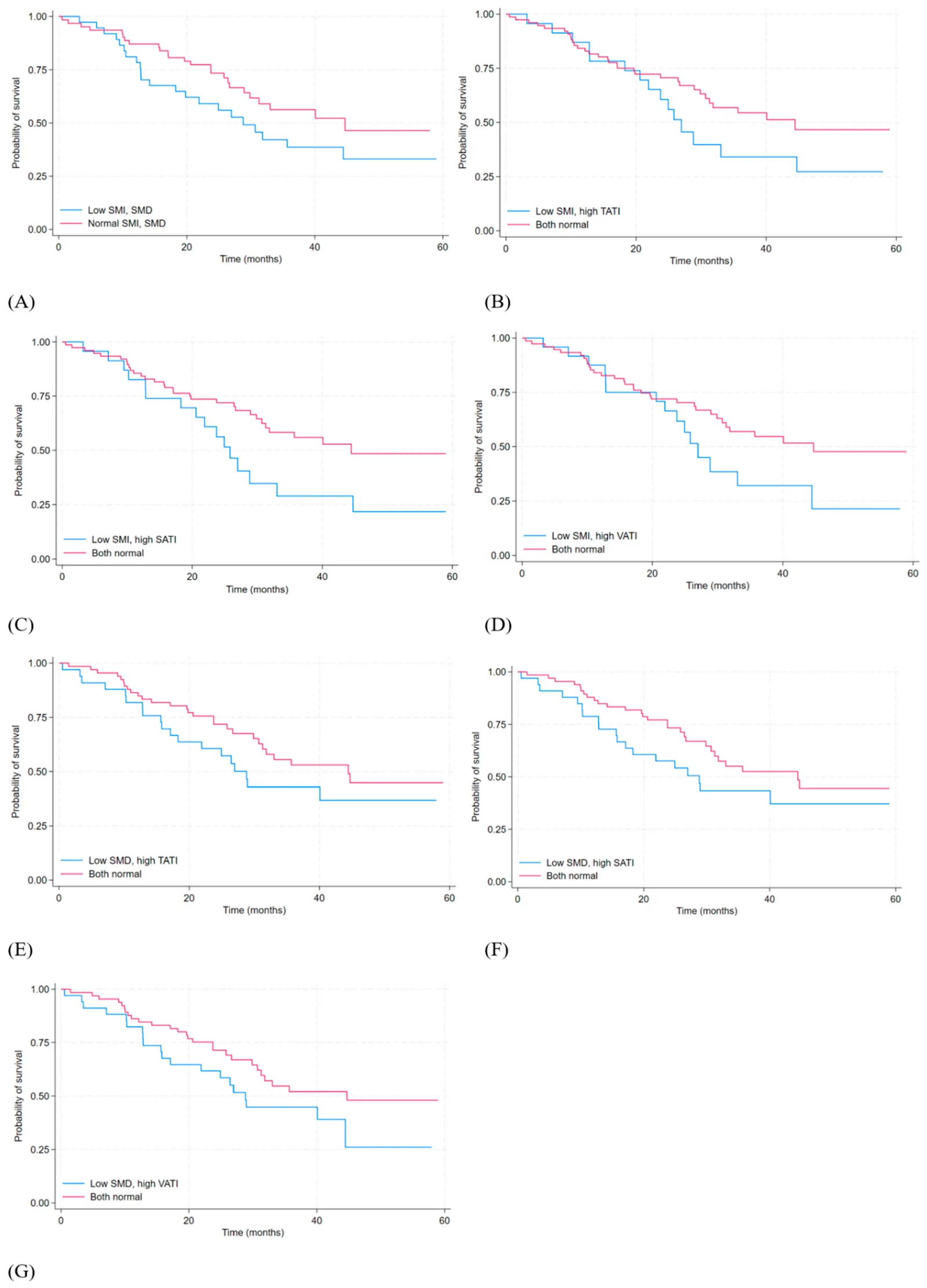
Kaplan–Meier survival curves for combined phenotypes: (**A**) low SMI and low SMD, (**B**) low SMI and high TATI, (**C**) low SMI and high SATI, (**D**) low SMI and high VATI, (**E**) low SMD and high TATI, (**F**) low SMD and high SATI, (**G**) low SMD and high VATI, SMI: skeletal muscle index, SMD: skeletal muscle density, TATI: total adipose tissue, SATI: subcutaneous adipose tissue index, VATI: visceral adipose tissue index.

The median follow-up time was 26 months, during which time 48 (48%) of the 99 included patients died. There was weak evidence that low SMD (HR: 1.74, 95% CI: 0.90–3.37, *p* = 0.098) and high VATI (HR: 1.87; 95% CI: 0.97–3.58; *p* = 0.060) as singular phenotypes were associated with a higher risk of mortality. There was insufficient evidence of an association between low SMI, high TATI or high SATI and mortality (Table 4). Among co-occurring phenotypes, there was moderate evidence of higher mortality in patients with low SMI with high TATI (HR: 1.91, 95% CI: 1.01–3.61; *p* = 0.046) and high SATI (HR: 2.03, 95% CI: 1.10–3.78; *p* = 0.025). There was weak evidence of higher mortality in patients with low SMI with high VATI (HR: 1.91, 95% CI: 0.98–3.72; *p* = 0.057). There was moderate evidence of higher mortality in patients with low SMD alongside high TATI (HR: 2.33, 95% CI: 1.03–5.28; *p* = 0.043) and high VATI (HR: 2.49, 95% CI: 1.10–5.67; *p* = 0.029) and weak evidence for higher mortality in patients with low SMD with high SATI (HR: 2.15, 95% CI: 0.99–4.67; *p* = 0.053). All results of the Cox proportional hazards analysis are shown in Table 4. Kaplan–Meier curves are presented for overall survival (Figure 3 and Figure 4).

## 4. Discussion

In this retrospective cohort study of 99 women with epithelial ovarian cancer (EOC), we found two-thirds of women had low muscle mass, over half had poor muscle composition, and only four women had normal body composition at the commencement of treatment. Low muscle mass in combination with high adiposity was associated with 1.91 to 2.03 times higher hazard of mortality, while low muscle density (reflecting muscle composition) in combination with high adiposity was associated with 2.15 to 2.49 times higher hazard of mortality. These associations, while exploratory, were independent of age, tumour stage, comorbidities and BMI. Interestingly, when single body composition phenotypes were examined, there was only moderate evidence of a higher hazard of mortality with low muscle density and high visceral adiposity. Although 45% experienced a postoperative complication, 13% were readmitted within 30 days, and 47% of those treated with neoadjuvant and 63% with adjuvant chemotherapy required a dose modification; there was no evidence of an association between these outcomes and body composition phenotype.

Throughout the literature in EOC, the influence of low SMI on survival is conflicting [17,18,19,20,21,22], and while low SMD is relatively novel, the association with survival is largely consistent [11,18,20,22,33]. However, binary investigations focusing on a single phenotype fail to recognise that body composition is multifaceted beyond skeletal muscle alone. Our analysis found that women with low SMI or low SMD who also had high adiposity had the highest risk of mortality. Conversely, as independent phenotypes, there was only weak or insufficient evidence of an association of low SMD and low SMI with mortality, suggesting the additive effect of adiposity is important. In this analysis, mortality risk was highest for women with low SMD co-occurring with high total and visceral adiposity. While the additive effects of low SMI co-occurring with high adiposity, referred to as sarcopenic obesity, have been identified as a unique phenotype associated with poor prognosis across numerous cancer types [53,54], the effects of low SMD co-occurring with high adiposity in both EOC and cancer more broadly are largely unexplored. In patients with head and neck cancer, Findlay et al. [55] did not detect any significant associations with survival for patients with low SMD and obesity (aHR: 0.79, 95% CI: 0.27–3.39, *p* = 0.70). However, Findlay et al. [55] used BMI to define obesity, potentially obscuring the true effect of adiposity. Although BMI is still widely utilised clinically, it is an imprecise measure of body composition, often misclassifying adipose tissue level due to its inability to distinguish skeletal muscle [26]. The importance of adiposity and need for precise evaluations of body composition are further underscored by our finding that low SMI was only associated with worse overall survival in the presence of high adiposity. These findings are aligned with four earlier studies using CT-based evaluations of body composition in EOC in samples ranging from 197 to 500 women with primarily advanced-stage disease. Collectively, these investigations showed high visceral fat-to-muscle [30], high total fat-to-muscle-ratio [31] and high adiposity co-occurring with low SMI [32], or low psoas indices [29] were associated with between a 1.55 and 3.3 increased hazard for mortality [29,30,31,32]. Despite the heterogeneity among the studies and psoas evaluations as a proxy for the muscle mass, together with our exploratory findings, these highlight the limitation of focusing on single phenotypes in ovarian cancer, which risks oversimplification.

Our results investigating associations between low SMI as a single phenotype aid in clarifying the discordant literature. Our finding that low SMI was not associated with survival is consistent with other recent studies in EOC [56,57] and aligned with meta-analyses that examined eight studies collectively [11,17,18,22]. Although, two additional meta-analyses reported low SMI was associated with a 30% increased hazard for mortality (95% CI: 1.07–1.58, *p* value not reported, *n* = 15 studies) [20] and 35% higher odds of mortality (95% CI: 1.05–1.74, *p* = 0.02, *n* = 8 studies) [21]; the conflicting findings from these analyses are likely attributable to methodological difference. In these analyses, studies evaluating SMI as a continuous variable, SMI from psoas or total SMA, and those with small sample sizes (*n* = 12) and unadjusted analyses were pooled together [20,21], and one meta-analysis used a different statistical approach (odds ratio) [21]. Our findings reinforce that in women with EOC, muscle mass must be assessed within the context of adipose tissue to adequately identify risk of adverse outcomes. For SMD as a single phenotype, in contrast to the existing body of literature both in EOC and across multiple diagnoses [58], our study showed only a weak association with survival (*p* = 0.098). In EOC, six meta-analyses collectively evaluating seven studies [11,17,18,20,22,33] have consistently reported strong associations between low SMD and survival. The lack of association in our study may, in part, be due to sample size; however, across this literature, there is substantial heterogeneity in methods used to define low SMD. In the absence of standardised cut points, most studies in EOC have utilised sample-derived definitions, including median, tertile, and mean-based methods ranging from <21.24 to <39 HU [59,60,61,62,63,64,65], and importantly, none were stratified by BMI. While the relevance of BMI-corrected cut points has been questioned due to its limitations in patients with ascites [61,65,66], lipid accumulation in skeletal muscle is associated with obesity [35], and SMD is negatively correlated with BMI [67]. One other study in women with predominantly advanced, high-grade EOC (*n* = 140) has used the same BMI-adjusted cut points by Martin et al. [44] to investigate associations with survival and reported no evidence of an association after adjustment for confounders (HR 1.44, 95% CI: 0.73–2.86, *p* = 0.297) [65]. However, when using a sample-derived cut point (<39 HU), SMD was an independent predictor for survival (HR: 2.25, 95% CI: 1.09–4.65, *p* = 0.028), highlighting the need to clarify clinically translatable definitions of low SMD.

In this study, 67% of women had low SMI, and 57% had poor muscle composition prior to commencing treatment. This is higher than estimates in previous studies of women with EOC. In 2022, a meta-analysis by Tranoulis et al. [17] found 41% had low SMI prior to treatment, with data pooled from fifteen studies including 2009 women with cut-off points ranging from 38.5 to 41.5 cm^2^/m^2^. Where reported, low SMD has ranged from 21 to 50% in samples of 140 to 323 women [11,20,33]. Differences in prevalence are likely explained by the definitions used to define low SMI and low SMD. In addition, we found 49% of women presented with high adiposity (based on the median) before treatment. Comparisons of the prevalence of high adiposity across the literature in EOC are limited by variations in the definition and cut points used for high adiposity, which encompass sample-dependent definitions using tertiles [32,64] or assessment of adipose tissue area [27,29,68] rather than index. In our cohort, only four women had normal body composition prior to commencing treatment, highlighting that for women with EOC, changes in body composition occur early in or prior to diagnosis, are pervasive, and represent a modifiable prognostic factor with important clinical implications.

A novel aspect of this study was the evaluation of body composition by menopausal status. We found moderate evidence that visceral adipose tissue distribution was associated with menopausal status, alongside weak evidence that high VATI was associated with poorer overall survival. Although EOC can occur at any age, most women are diagnosed post menopause (85% in this study), and this life stage is largely characterised by a shift in adipose tissue distribution, irrespective of body weight changes, with an observed increase in visceral fat [34,69]. This disproportionate shift also represents a transition to a metabolically unfavourable phenotype. Adipose tissue is an active endocrine and immunomodulatory organ with distinct patterns of adipokine secretion between subcutaneous and visceral tissue compartments [70,71,72]. The accumulation of visceral adipose tissue is characterised by dysregulated production of pro-inflammatory adipokines and immune activation, which may influence both tumour progression and the pharmacokinetics and pharmacodynamics of anti-cancer therapies [69,70,73]. Our finding that muscle mass and composition were not associated with menopausal status was unexpected, given the research showing accelerated gains in fat mass with concurrent loss of lean mass are a menopause-related phenomenon [74]. In this cohort, only 16 women were premenopausal at diagnosis, which may not be sufficient to detect true differences in skeletal muscle morphology and also warrants cautious interpretation of visceral adiposity differences, which require further validation. However, most women in this study also had an advanced stage of disease (83%); therefore, it is difficult to distinguish menopause-related from cancer-related changes in muscle morphology. Whereas, the redistribution of adipose tissue with increased accumulation of visceral fat is not commonly associated with the cancer-induced pro-catabolic shift in metabolism [75].

Although 45% of women in this study who underwent surgery experienced a postoperative complication, and 47% of those treated with neoadjuvant and 63% treated with adjuvant chemotherapy required a dose reduction, we found no evidence of an association of these outcomes with body composition. This may be a reflection of sample size and the number of events recorded. Notably, these findings are aligned with two earlier systematic reviews collectively evaluating postoperative complications in six studies in EOC [18,22], and more recently, two studies with 298 [76] and 192 [77] women, reporting no associations between low SMI and postoperative complications. While two other meta-analyses in EOC, with four additional studies, have reported low muscle mass was associated with increased complication risk, these analyses pooled studies with psoas evaluations and unadjusted results, which may explain the discrepancy in these findings [17,20]. Less research has investigated the influence of muscle composition and adipose tissue on postoperative complications in EOC. Evidence from a 2023 meta-analysis [20] and 2024 systematic review [78], together evaluating five original studies in EOC (samples ranging from 62 to 111) suggest poor muscle composition may be a risk factor for postoperative complications. While the results of our study are not aligned with these findings, sample-based definitions of low SMD in these studies may explain differences. Four studies with samples ranging from 61 to 298 women have evaluated the effect of adiposity on postoperative complications in EOC [68,76,77,79], with only one study detecting any association [76]. In this study by Heus et al. [76] (*n* = 298 women with advanced EOC), visceral obesity, defined as >100 cm^2^, was associated with a higher occurrence of postoperative complications. In colorectal cancer surgery, a recent meta-analysis of 28 studies with 11,129 patients reported high visceral adiposity was associated with higher rates of postoperative complications (OR: 1.96, 95% CI: 1.22–1.96, *p* = 0.003) [80]. Another single study on colorectal cancer (*n* = 1630) evaluating SMD reported that low preoperative muscle composition was associated with higher odds of having a major postoperative complication (OR: 2.42, 95% CI: 1.44–4.04) [37]. Increased lipid accumulation in muscle is associated with higher frailty and functional impairments, which are important factors for preoperative risk assessment [58]. In addition, elevated visceral adiposity increases surgical complexity and together contribute to local and systemic inflammation, potentially aggravating the surgically induced metabolic response, compromising recovery and wound healing [58,80]. In EOC, the achievement of optimal cytoreduction remains the strongest independent prognostic factor for survival, which often necessitates complex and aggressive procedures [6] and increasing risk of postoperative complications [81]. Preoperative risk prediction tools are critically important in major abdominal procedures, particularly among high-risk patients [82]. These findings highlight that in EOC, muscle composition may be more important than muscle quantity, and that the distribution of adipose tissue may be more important than the amount. While this research remains in its infancy, more detailed assessments of body composition could hold potential for enhanced personalisation of preoperative risk assessment and access to early interventions.

Combination chemotherapy with taxane and platinum-based drugs are important first-line treatments for EOC [6]. Traditionally, body surface area (BSA) is utilised to determine the dosage and prescription of systemic anti-cancer treatments [83]. One of the challenges with chemotherapy dosing is balancing treatment efficacy with treatment tolerability [84]. As our understanding of body composition has evolved, the continued use of BSA-based dosing has been questioned [83]. The potential for harnessing more detailed measures of body composition for chemotherapy dosing was recently demonstrated by Assenat et al. [85] in patients with colon cancer. In this investigation, chemotherapy was dosed according to muscle mass rather than BSA in patients with low muscle mass, resulting in reduced oxaliplatin-induced peripheral neuropathy without compromising efficacy [85]. In EOC, chemotherapy-induced peripheral neuropathy is one of the most important dose-limiting toxicities experienced by women [86]. In 2019, an international survey with 1360 women treated for ovarian cancer found obesity was independently associated with higher rates of neuropathy [87], while two retrospective analyses in EOC with 192 [88] and 105 [89] medical records reported 45% and 48% of women required a modification, with one study finding modifications were associated with worse overall survival (HR: 2.09, 95% CI: 1.24–3.51, *p* = 0.05) [88]. Similarly, it has been suggested that higher adiposity may reduce the efficacy of antiangiogenic agents such as bevacizumab; however, a study in 218 women with EOC did not find adiposity to be associated with reduced treatment efficacy [90]. Therefore, any potential for enhancing chemotherapy tolerance with more sophisticated and personalised dosing strategies is clinically meaningful. A systematic review by Rizzo et al. [84] with six studies in EOC (*n* = 761) found body composition was associated with cycle delays, neuropathy, grade 3 toxicities, toxicity-induced modifications to treatment and early discontinuation of chemotherapy. However, these studies varied substantially in body composition compartments assessed. In this review, SMA was associated with chemotherapy discontinuation [91], whereas SMI was not associated with any chemotherapy-related outcomes [91,92]. Muscle composition and adipose tissue were associated with cycle delays and toxicity severity [59,91], while fat-to-muscle ratio was associated with toxicity in overweight and obese patients only [93]. Another retrospective study with 173 medical records of women treated for advanced-stage ECO found that patients with low SMI (<39 cm^2^/m^2^) were less likely to complete the recommended six cycles of chemotherapy [94], and the mean relative dose intensity was lower for patients with low muscle mass compared to those with normal muscle mass. Although our study did not detect any associations between body composition and chemotherapy-related outcomes, additional research is needed to clarify the effect of body composition on chemotherapy drug distribution, both to improve survival outcomes and to enhance quality of life during intensive treatment.

The strengths of this study include the evaluation of all body composition compartments available for assessment on a CT scan. Our analysis also considered the differential and combined effects of co-occurring phenotypes, where sample size permitted, and the effect of menopausal status on body composition. Limitations include the retrospective nature of the study, which relied on data reported in the medical record. This investigation was a single-centre evaluation and likely influenced by selection bias, given pretreatment CT images were required for inclusion. Although multivariable models were used, the sample size (e.g., 99 observations total with 48 deaths during the study period) limited statistical power and the number of confounders that could be adjusted for in the analysis. These limitations should be considered when interpreting the findings.

## 5. Conclusions

In summary, in this cohort of patients with ovarian cancer, only four women had normal body composition prior to commencing treatment. Of the phenotypes evaluated, evidence of an association with poorer overall survival was greatest for women with low muscle mass or poor muscle composition who also had high adiposity, highlighting the complexity of body composition. Post-menopausal women were more likely to present with a metabolically unfavourable phenotype characterised by high visceral adiposity. Although our study did not detect any evidence of an association between surgical and chemotherapy-related outcomes with body composition, additional research is warranted to clarify whether more sophisticated evaluations of body composition could enhance chemotherapy dosing methods to improve efficacy and tolerability and better personalise preoperative risk stratification. Overall, leveraging routinely acquired CT images to comprehensively quantify body composition may enable more precise risk stratification across treatment modalities and support personalised oncology care. Future studies should continue to consider the multifaceted nature of body composition and the potentially additive effects of co-occurring phenotypes to adequately capture high-risk phenotypes and inform clinical practice.

## Figures and Tables

**Table 1:** Demographic and clinical characteristics (*N* = 99).

Characteristic	Mean ± SD or *n* (%)
Age at diagnosis	61 ± 12.7
Height (cm)	161.0 ± 7.1
Weight (kg)	71 ± 21.3
BMI (kg/m^2^)	27.4 ± 7.9
BMI category (kg/m^2^)	
<18.5	4 (4%)
18.5–24.9	42 (42%)
25.0–29.9	29 (29%)
≥30 kg/m^2^	24 (24%)
SMI cm^2^/m^2^	38.9 ± 7.2
SMD HU	35.8 ± 10.7
Menopause ^a^	
Post menopause	80 (83%)
Pre menopause	16 (17%)
ECOG ^b^	
Not impaired ≤ 1	84 (87%)
Impaired ≥ 2	13 (13%)
CCI, median [IQR] ^c^	2.4 [1-3]
Stage ^d^	
Early (I/II)	16 (17%)
Advanced (III/IV)	80 (83%)
Histology ^e^	
High grade serous	66 (67%)
Low grade serous	5 (5%)
Endometrioid	4 (4%)
Clear cell	11 (11%)
Mucinous	6 (6%)
Other	1 (1%)
Mixed histology	5 (5%)
Ascites ^f^	
No ascites	44 (46%)
Ascites	52 (54%)
Treatment characteristics	
Surgery	75 (76%)
Primary debulking	25 (33%)
Primary debulking with IPC	1 (1%)
Interval debulking	41 (55%)
Interval debulking with IPC	8 (11%)
Residual disease	66 (88%)
Complete (no macroscopic disease)	33 (50%)
Optimal (<1 cm)	23 (35%)
Suboptimal (≥1 cm)	10 (15%)
Chemotherapy	76 (77%)
Neoadjuvant only	24 (24%)
Adjuvant only	19 (19%)
Both schedules	33 (33%)
Targeted therapy	32 (32%)
Radiotherapy	3 (3%)
Hormone therapy	6 (6%)
Body composition phenotype(s)	
Low SMI (<41 cm^2^/m^2^)	67 (67%)
Low SMD (HU) ^g^	57 (57%)
High TATI (≥107.9 cm^2^/m^2^) ^h^	49 (49%)
High SATI (≥80.5 cm^2^/m^2^) ^h^	49 (49%)
High VATI (≥30.1 cm^2^/m^2^) ^h^	49 (49%)
Co-occurring phenotypes	
Low SMI + low SMD	37 (37%)
*Low SMI + high adiposity*	
Low SMI + High TATI	23 (23%)
Low SMI + High SATI	23 (23%)
Low SMI + High VATI	24 (24%)
*Low SMD + high adiposity*	
Low SMD + High TATI	33 (33%)
Low SMD + High SATI	33 (33%)
Low SMD + High VATI	34 (34%)
*All phenotypic combinations*	
Low SMI + Low SMD + High TATI	15 (15%)
Low SMI + Low SMD + High SATI	16 (16%)
Low SMI + Low SMD + High VATI	16 (16%)

Abbreviations: BMI: body mass index, CCI: Charlson Co-morbidity Index, ECOG: Eastern Cooperative Oncology Group performance status score, IPC: intraperitoneal chemotherapy, SMD: skeletal muscle density, SMI: skeletal muscle index, SATI: subcutaneous adipose tissue index, TATI: total adipose tissue index, VATI: visceral adipose tissue index. ^a^ Menopause *n* = 96, data missing for three records, ^b^ ECOG, *n* = 97, date missing for two records, ^c^ CCI, *n* = 98, data missing for one record, ^d^ Stage, *n* = 96, three records missing, ^e^ Histology, *n* = 98, one record missing, ^f^ Ascites, *n* = 96, three records missing. ^g^ SMD cut off points: <41 HU for women BMI < 24.5 kg/m^2^, <33 HU for women BMI > 25.0 kg/m^2^. ^h^ Adipose tissue analysis not available for one record, *n* = 98. Median-based cut points for adipose tissues, TATI: ≥107.9 cm^2^/m^2^, SATI: ≥80.5 cm^2^/m^2^, VATI: ≥30.1 cm^2^/m^2^.

**Table 2:** Association between patient and clinical characteristics and pretreatment body composition.

Characteristic	SMI (*n* = 99) *n* (%)			SMD (*n* = 99) *n* (%)			TATI (*n* = 98) *n* (%)		
	Normal	Low	*p* Value	Normal	Low	*p* Value	Normal	High	*p* Value
Age	62 (11.0)	60 (13.5)	0.574	57 (12.8)	63 (12)	0.012	59 (14.8)	62 (9.8)	0.134
Height (cm)	160.1 (6.4)	161.5 (7.4)	0.361	162.1 (6.8)	160.3 (7.3)	0.196	161.3 (7.0)	160.8 (7.3)	0.728
Weight (kg)	88.2 (25.6)	62.9 (12.5)	<0.001	66.1 (14.0)	74.7 (25.0)	0.048	58.2 (8.8)	84.3 (22.2)	<0.001
BMI, mean (SD)	34.3 (9.2)	24.0 (4.3)	<0.001	25.0 (4.5)	29.1 (9.4)	0.012	22.3 (2.6)	32.6 (8.1)	<0.001
Menopause ^a^									
Post menopause	28 (30%)	52 (54%)		32 (33%)	48 (50%)		35 (36%)	44 (46%)	
Pre menopause	4 (4%)	9 (13%)	0.567	9 (9%)	7 (7%)	0.230	11 (11%)	5 (5%)	0.074
ECOG ^b^									
Not impaired ≤ 1	28 (29%)	56 (58%)		40 (41%)	44 (45%)		42 (44%)	42 (44%)	
Impaired ≥ 2	4 (4%)	9 (9%)	1.0	1 (1%)	12 (12%)	0.006	5 (5%)	7 (7%)	0.589
CCI, mean (SD) ^c^	2.8 (2.0)	2.2 (1.6)	0.118	1.8 (1.5)	2.8 (1.8)	0.057	2.0 (1.5)	2.8 (1.9)	0.017
Stage ^d^									
Early (I/II)	7 (7%)	9 (9%)		8 (8%)	8 (8%)		5 (5%)	11 (12%)	
Advanced (III/IV)	24 (25%)	56 (58%)	0.283	34 (35%)	46 (56%)	0.581	42 (44%)	37 (39%)	0.110
Histology ^e^									
High grade serous	20 (20%)	46 (47%)		27 (28%)	39 (40%)		32 (33%)	33 (34%)	
All others	11 (11%)	21 (21%)	0.684	15 (15%)	17 (17%)	0.576	17 (18%)	15 (15%)	0.718
Ascites ^f^									
No ascites	16 (17%)	28 (29%)		19 (20%)	25 (26%)		19 (20%)	25 (26%)	
Ascites present	16 (17%)	36 (38%)	0.562	21 (22%)	31 (32%)	0.782	28 (29%)	23 (24%)	0.255

Abbreviations: BMI: body mass index, CCI: Charlson Co-morbidity Index, ECOG: Eastern Cooperative Oncology. Group performance status score, SMD: skeletal muscle density, SMI: skeletal muscle index, TATI: total adipose tissue index. ^a^ Menopause sample *n* = 96 for SMI and SMD, *n* = 95 for TATI, ^b^ ECOG sample *n* = 97 for SMI and SMD, *n* = 96 for TATI, ^c^ CCI sample *n* = 98 for SMI and SMD, *n* = 97 for TATI, ^d^ Stage sample *n* = 96 for SMI and SMD, *n* = 95 for TATI, ^e^ Histology sample *n* = 98 for SMI and SMD, *n* = 97 for TATI, ^f^ Ascites sample *n* = 96 for SMI and SMD, *n* = 95 for TATI.

**Table 3:** Associations between pretreatment body composition and hospitalisation, complication severity and chemotherapy-related outcomes.

Phenotype	Hospitalisation				Complications			Neoadjuvant Chemotherapy				Adjuvant Chemotherapy					
	LOS (*n* = 75) Median [IQR]	*p* Value	30-Day Readmission (*n* = 10) *n*%	*p* Value	Minor (*n* = 23) *n*%	Severe (*n* = 11) *n*%	*p* Value	NACT Toxicity (*n* = 42) *n*%	*p* Value	NACT Dose Modification (*n* = 27) *n*%	*p* Value	Time to Adjuvant CT (*n* = 52) Median [IQR]	*p* Value	Adjuvant CT Toxicity (*n* = 31) *n*%	*p* Value	Adjuvant CT Dose Modification (*n*= 33) *n*%	*p* Value
Muscle																	
Low SMI	3.5 [2-7]		7 (9%)		18 (53%)	6 (18%)		28 (51%)		18 (32%)		23 [21-28]		24 (46%)		24 (47%)	
Normal	3 [2-7]	0.879	3 (4%)	1.0	5 (15%)	5 (15%)	0.156	14 (25%)	0.734	9 (16%)	0.928	28 [21-51]	0.161	7 (13%)	0.226	9 (18%)	0.650
Low SMD	3 [2-7]		5 (7%)		15 (44%)	6 (18%)		23 (42%)		17 (30%)		23 [21-26]		14 (27%)		16 (31%)	
Normal	3.5 [2-8]	0.856	5 (7%)	0.615	8 (24%)	5 (15%)	0.549	19 (35%)	0.522	10 (18%)	0.396	27 [21–30]	0.138	17 (33%)	0.397	17 (33%)	0.629
SMI + SMD																	
Both low	3 [2-7]		4 (5%)		12 (35%)	3 (8%)		15 (27%)		11 (20%)		21 [20-25]		11 (21%)		12 (24%)	
Normal	3.5 [2-8]	0.451	6 (8%)	1.0	11 (32%)	8 (24%)	0.271	27 (49%)	0.498	16 (29%)	0.629	27 [21–31]	0.050	20 (38%)	0.592	21 (41%)	0.572
Adipose tissue																	
High TATI	3 [2-6]		8 (11%)		12 (35%)	7 (21%)		20 (37%)		11 (20%)		23 [21-31.5]		20 (38%)		19 (37%)	
Normal/low	4 [3-9]	0.111	2 (3%)	0.086	11 (32%)	4 (12%)	0.715	21 (76%)	0.750	15 (27%)	0.227	25 [21-29]	0.988	11 (21%)	0.592	14 (27%)	0.525
High SATI	3 [2-6]		7 (9%)		10 (29%)	7 (21%)		19 (35%)		11 (20%)		25 [21-29]		20 (38%)		21 (41%)	
Normal/low	3.5 [2-9]	0.328	3 (4%)	0.309	13 (38%)	4 (12%)	0.465	22 (41%)	0.637	15 (27%)	0.341	22 [21–28]	0.642	11 (21%)	0.382	12 (24%)	0.344
High VATI	3 [2-6]		7 (9%)		11 (32%)	6 (18%)		17 (31%)		10 (18%)		21 [21-33]		22 (42%)		18 (35%)	
Normal/low	4 [3-9]	0.111	3 (4%)	0.174	12 (35%)	5 (15%)	0.714	24 (44%)	0.434	16 (29%)	0.324	23 [21-27.5]	0.288	9 (17%)	0.089	15 (29%)	0.217
Combined phenotypes Low SMI + adipose tissues																	
Low SMI + high TATI	4 [3-9]		-	-	7 (21%)	2 (6%)		8 (15%)		6 (11%)		22.5 [21-35]		7 (13%)		8 (16%)	
Normal	3 [2-7]	0.318	-	-	16 (47%)	9 (26%)	0.682	34 (62%)	0.709	21 (38%)	0.639	25 [21-28.5]	0.966	24 (46%)	0.721	25 (49%)	0.462
Low SMI + high SATI	4 [2-6]		-	-	9 (26%)	1 (3%)		10 (18%)		7 (13%)		21 [21-25]		8 (15%)		7 (14%)	
Normal	3 [2-8]	0.299	-	-	14 (41%)	10 (29%)	0.113	32 (58%)	1.0	20 (36%)	0.643	26 [21-30]	0.266	23 (44%)	0.491	26 (51%)	1.0
Low SMI + high VATI	4 [2-7]		-	-	8 (24%)	2 (6%)		11 (20%)		8 (14%)		22 [21-26]		6 (12%)		8 (16%)	
Normal	3 [2-7.5]	0.510	-	-	15 (44%)	9 (26%)	0.437	31 (56%)	1.0	19 (33%)	0.440	25 [21-30.5]	0.259	25 (48%)	1.0	25 (49%)	0.462
Low SMD + adipose tissues																	
Low SMD + high TATI	4 [2-7]		-	-	9 (26%)	3 (8%)		12 (22%)		9 (16%)		23 [21-35]		6 (12%)		8 (16%)	
Normal	3 [2-7]	0.885	-	-	14 (41%)	8 (24%)	0.705	30 (55%)	1.0	18 (32%)	0.447	25 [21-29]	0.803	25 (48%)	0.700	25 (49%)	0.726
Low SMD + high SATI	4.5 [2-7]		-	-	11 (32%)	3 (9%)		13 (24%)		9 (16%)		21 [21-28]		5 (10%)		6 (12%)	
Normal	3 [2-7]	0.527	-	-	12 (35%)	8 (24%)	0.295	29 (53%)	1.0	18 (32%)	0.640	25 [21-30]	0.454	26 (50%)	0.281	27 (53%)	0.488
Low SMD + high VATI	4 [2-7]		-	-	10 (29%)	3 (9%)		14 (25%)		11 (20%)		23 [21-27]		6 (12%)		9 (18%)	
Normal	3 [2-7.5]	0.821	-	-	13 (38%)	8 (24%)	0.465	28 (51%)	1.0	16 (29%)	0.184	25 [21-30]	0.562	25 (48%)	0.439	24 (47%)	0.502
All phenotypes: low SMI + low SMD + adipose tissue																	
Low SMI, low SMD + high TATI	5 [2-7]		-	-	6 (18%)	1 (3%)		4 (7%)		3 (5%)		24 [21-35]		4 (8%)		5 (10%)	
Normal	3 [2-7]	0.891	-	-	17 (50%)	10 (29%)	0.384	38 (69%)	0.619	24 (43%)	1.0	24.5 [21-28.5]	0.698	27 (52%)	1.0	28 (55%)	0.405
Low SMI, low SMD + high SATI	5 [2-6.5]		-	-	8 (24%)	1 (3%)		6 (11%)		4 (7%)		21 [21-25]		4 (8%)		4 (8%)	
Normal	3 [2-7]	0.874	-	-	15 (44%)	10 (29%)	0.214	36 (65%)	1.0	23 (41%)	1.0	25 [21-30]	0.373	27 (52%)	1.0	29 (57%)	0.686
Low SMI, low SMD + high VATI	4.5 [2-6.5]		-	-	7 (21%)	1 (3%)		6 (11%)		5 (9%)		23 [21-26]		4 (8%)		6 (12%)	
Normal	3 [2-7]	0.982	-	-	16 (47%)	10 (29%)	0.227	36 (65%)	1.0	22 (39%)	0.462	25 [21-30]	0.522	27 (52%)	1.0	27 (53%)	0.398

Abbreviations: CT: chemotherapy, LOS: length of stay, NACT: neoadjuvant chemotherapy, SMD: skeletal muscle density, SMI: skeletal muscle index, TATI: total adipose tissue index.

**Table 4:** Association between pretreatment body composition and overall survival.

Phenotype	Death (*n/N*) ^a^	Hazard Ratio (95% CI)	*p*-Value
Low SMI	32/64	1.36 (0.68–2.74)	*p* = 0.389
Normal	12/31	1.0 (reference)	
Low SMD ^a^	30/54	1.74 (0.90–3.37)	*p* = 0.098
Normal	14/41	1.0 (reference)	
High TATI	26/48	1.70 (0.89–3.25)	*p* = 0.109
Normal	17/46	1.0 (reference)	
High SATI	26/48	1.65 (0.86–3.17)	*p* = 0.129
Normal	17/46	1.0 (reference)	
High VATI	25/46	1.87 (0.97–3.58)	*p* = 0.060
Normal	18/48	1.0 (reference)	
Combined phenotypes			
Muscle			
Low SMI + Low SMD	21/35	1.68 (0.88–3.19)	*p* = 0.115
Normal	23/60	1.0 (reference)	
SMI + adipose tissues			
Low SMI + High TATI	15/23	1.91 (1.01–3.61)	*p* = 0.046
Normal	29/72	1.0 (reference)	
Low SMI + High SATI	16/23	2.03 (1.10–3.78)	*p* = 0.025
Normal	28/72	1.0 (reference)	
Low SMI + High VATI	14/22	1.91 (0.98–3.72)	*p* = 0.057
Normal	30/73	1.0 (reference)	
SMD + adipose tissues			
Low SMD + High TATI	18/32	2.33 (1.03–5.28)	*p* = 0.043
Normal	26/63	1.0 (reference)	
Low SMD + High SATI	18/32	2.15 (0.99–4.67)	*p* = 0.053
Normal	26/63	1.0 (reference)	
Low SMD + High VATI	18/31	2.49 (1.10–5.67)	*p* = 0.029
Normal	26/64	1.0 (reference)	

Models adjusted for age, stage and CCI. SMD model also adjusted for BMI. ^a^ Total “*N*” varies based on missing covariate data.

## Data Availability

The de-identified data collected in this study was obtained from a retrospective analysis of medical records and routine CT imaging. Due to institutional governance and privacy restrictions, the data are not publicly available. The data may be made available upon request to the corresponding author, and subject to approval from the relevant hospital governance and ethics committees.

## Supplementary Materials

The following supporting information can be downloaded at  https://www.mdpi.com/article/10.3390/cancers18091478/s1. Table S1: Directed acyclic graphs (DAGitty graphs) of proposed relationships between body composition variable (exposure) and survival and postoperative complications(outcomes). Figure S1: Directed acyclic graphs (DAGitty graphs) of proposed relationships between body composition variable (exposure) and survival and postoperative complications (outcomes).

## Abbreviations

CT	Computed Tomography
BMI	Body Mass Index
SMD	Skeletal Muscle Density
SMI	Skeletal Muscle Index
EOC	Epithelial Ovarian Cancer
EMR	Electronic Medical Records
CCI	Charlson Co-Morbidity Score
ECOG	Eastern Cooperative Oncology Group (ECOG) performance status
PDS	Primary Debulking Surgery
IDS	Interval Debulking Surgery
SMA	Skeletal Muscle Area
VAT	Visceral Adipose Tissue
SAT	Subcutaneous Adipose Tissue
TATI	Total Adipose Tissue
SATI	Subcutaneous Adipose Tissue Area
VATI	Visceral Adipose Tissue Area
CTCAE	Common Terminology Criteria for Adverse Events
LOS	Length of Stay
BSA	Body Surface Area
