# Correction: Benna-Doyle et al. Investigating the Prognostic Value of Pretreatment Body Composition in Women with Ovarian Cancer: Impact on Clinical Outcomes. *Cancers* 2026, *18*, 1478

**DOI:** 10.3390/cancers18132055

**Published:** 2026-06-25

**Authors:** Sarah Benna-Doyle, Erin Laing, Brenton J. Baguley, Nicholas Hardcastle, Gavin Abbott, Nicole Kiss

**Affiliations:** 1 Institute for Physical Activity and Nutrition, Deakin University, Geelong, VIC 3216, Australia; b.baguley@deakin.edu.au (B.J.B.); gavin.abbott@deakin.edu.au (G.A.); nicole.kiss@deakin.edu.au (N.K.); 2 School of Exercise and Nutrition Sciences, Deakin University, Burwood, VIC 3125, Australia; erin.laing@petermac.org; 3 Nutrition and Speech Pathology Department, Peter MacCallum Cancer Centre, Melbourne, VIC 3000, Australia; 4 Sir Peter MacCallum Department of Oncology, University of Melbourne, Melbourne, VIC 3000, Australia; nick.hardcastle@petermac.org; 5 Department of Radiation Oncology, Peter MacCallum Cancer Centre, Melbourne, VIC 3000, Australia

## Error in Table

In the original publication [1], there was a mistake in Table 2 as published. The percentage of patients with high-grade serous histology and low skeletal muscle density (SMD) was incorrectly reported as 57%. The correct percentage is 40%. The corrected Table 2 appears below. The authors state that the scientific conclusions are unaffected. This correction was approved by the Academic Editor. The original publication has also been updated.

## Figures and Tables

**Table 2:** Association between patient and clinical characteristics and pretreatment body composition.

Characteristic	SMI (*n* = 99) *n* (%)			SMD (*n* = 99) *n* (%)			TATI (*n* = 98) *n* (%)		
	Normal	Low	*p* Value	Normal	Low	*p* Value	Normal	High	*p* Value
Age	62 (11.0)	60 (13.5)	0.574	57 (12.8)	63 (12)	0.012	59 (14.8)	62 (9.8)	0.134
Height (cm)	160.1 (6.4)	161.5 (7.4)	0.361	162.1 (6.8)	160.3 (7.3)	0.196	161.3 (7.0)	160.8 (7.3)	0.728
Weight (kg)	88.2 (25.6)	62.9 (12.5)	<0.001	66.1 (14.0)	74.7 (25.0)	0.048	58.2 (8.8)	84.3 (22.2)	<0.001
BMI, mean (SD)	34.3 (9.2)	24.0 (4.3)	<0.001	25.0 (4.5)	29.1 (9.4)	0.012	22.3 (2.6)	32.6 (8.1)	<0.001
Menopause ^a^									
Post menopause	28 (30%)	52 (54%)		32 (33%)	48 (50%)		35 (36%)	44 (46%)	
Pre menopause	4 (4%)	9 (13%)	0.567	9 (9%)	7 (7%)	0.230	11 (11%)	5 (5%)	0.074
ECOG ^b^									
Not impaired ≤ 1	28 (29%)	56 (58%)		40 (41%)	44 (45%)		42 (44%)	42 (44%)	
Impaired ≥ 2	4 (4%)	9 (9%)	1.0	1 (1%)	12 (12%)	0.006	5 (5%)	7 (7%)	0.589
CCI, mean (SD) ^c^	2.8 (2.0)	2.2 (1.6)	0.118	1.8 (1.5)	2.8 (1.8)	0.057	2.0 (1.5)	2.8 (1.9)	0.017
Stage ^d^									
Early (I/II)	7 (7%)	9 (9%)		8 (8%)	8 (8%)		5 (5%)	11 (12%)	
Advanced (III/IV)	24 (25%)	56 (58%)	0.283	34 (35%)	46 (56%)	0.581	42 (44%)	37 (39%)	0.110
Histology ^e^									
High grade serous	20 (20%)	46 (47%)		27 (28%)	39 (40%)		32 (33%)	33 (34%)	
All others	11 (11%)	21 (21%)	0.684	15 (15%)	17 (17%)	0.576	17 (18%)	15 (15%)	0.718
Ascites ^f^									
No ascites	16 (17%)	28 (29%)		19 (20%)	25 (26%)		19 (20%)	25 (26%)	
Ascites present	16 (17%)	36 (38%)	0.562	21 (22%)	31 (32%)	0.782	28 (29%)	23 (24%)	0.255

Abbreviations: BMI: body mass index, CCI: Charlson Co-morbidity Index, ECOG: Eastern Cooperative Oncology. Group performance status score, SMD: skeletal muscle density, SMI: skeletal muscle index, TATI: total adipose tissue index. ^a^ Menopause sample *n* = 96 for SMI and SMD, *n* = 95 for TATI, ^b^ ECOG sample *n* = 97 for SMI and SMD, *n* = 96 for TATI, ^c^ CCI sample *n* = 98 for SMI and SMD, *n* = 97 for TATI, ^d^ Stage sample *n* = 96 for SMI and SMD, *n* = 95 for TATI, ^e^ Histology sample *n* = 98 for SMI and SMD, *n* = 97 for TATI, ^f^ Ascites sample *n* = 96 for SMI and SMD, *n* = 95 for TATI.
