# Supplementary material for: Investigating the Prognostic Value of Pretreatment Body Composition in Women with Ovarian Cancer: Impact on Clinical Outcomes
Source: Cancers (Basel). 2026 May 4;18(9):1478. doi: 10.3390/cancers18091478 (PMC13162712; doi:10.3390/cancers18091478)
Supplement: Supplementary file 1 [file cancers-18-01478-s001.zip › BennaDoyle_Cancer Special Issue_Supplementary material_27 March 2026.pdf]

Figure S1: Directed acyclic graphs (DAGitty graphs) of proposed relationships between body composition variable (exposure) and survival and postoperative complications(outcomes).

DAGitty graphs for survival:

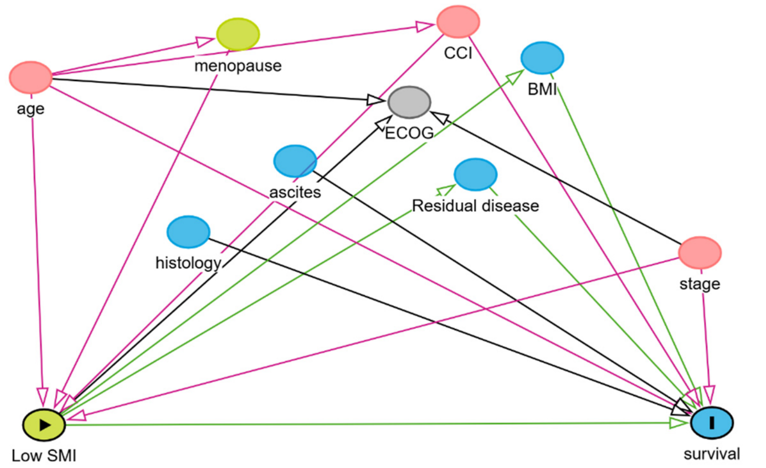

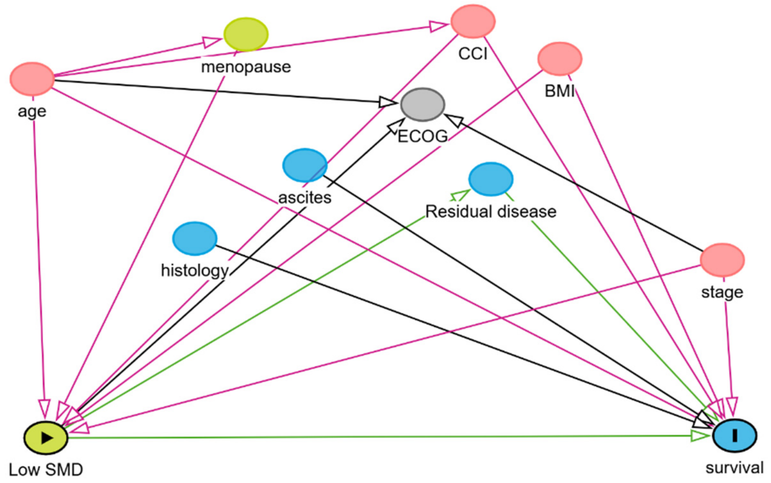

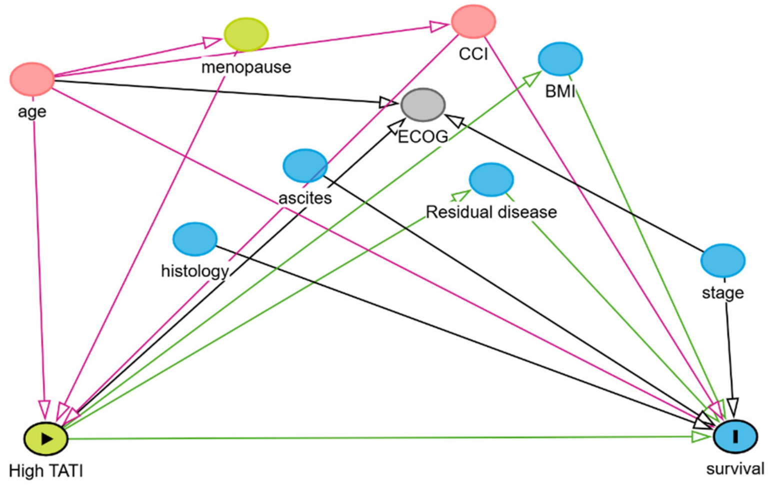

Dagitty graphs for postoperative complications:

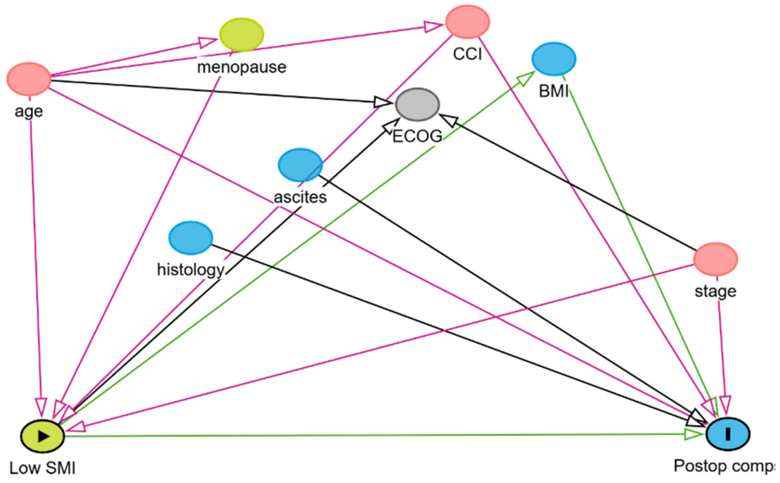

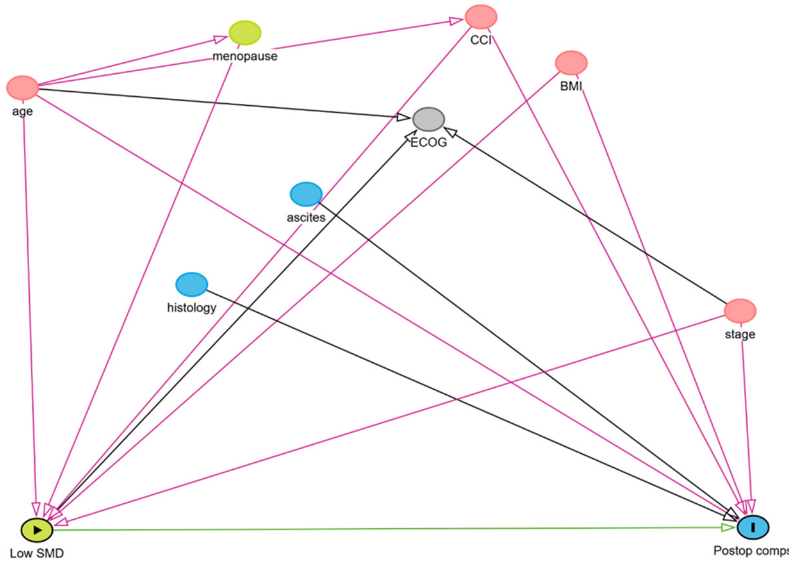

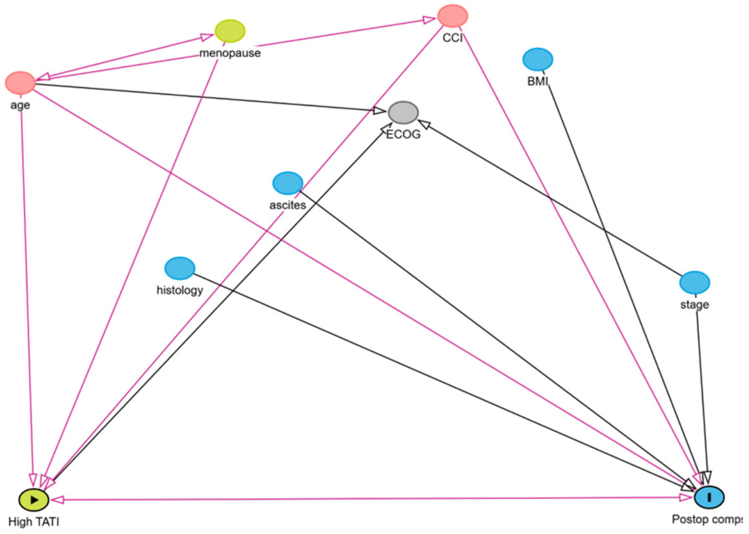

Table S1. Association between pretreatment body composition and postoperative complications

Phenotype	Postoperative complication (*n/N*)	Odds ratio (95%CI)	*P*-value
Low SMI	24/50	1.42 (.52 - 3.86)	p =0.494
Normal	10/25	1.0 (reference)	
Low SMD	21/43	1.18 (.45 - 3.13)	p = 0.734
Normal	13/32	1.0 (reference)	
Low SMI + Low SMD	15/27	1.74 (.65 - 4.68)	p =0.274
Both normal	19/48	1.0 (reference)	
High TATI	15/37	0.59 (.22 - 1.55)	p = 0.287
Normal	19/38	1.0 (reference)	
High SATI	17/37	1.07 (.42 - 2.72)	p = 0.877
Normal	17/38	1.0 (reference)	
High VATI	17/40	0.53 (.20 - 1.46)	p = 0.223
Normal	17/35	1.0 (reference)	
Low SMI + High TATI	9/17	1.36 (.45- 4.13)	p = 0.591
Both normal	25/58	1.0 (reference)	
Low SMI + High SATI	10/17	2.06 (.67 – 6.30)	p = 0.206
Both normal	24/58	1.0 (reference)	
Low SMI + High VATI	10/19	1.10 (.36 - 3.39)	p = 0.863
Both normal	24/56	1.0 (reference)	
Low SMD + High TATI	12/26	0.87 (.31 - 2.41)	p = 0.787
Both normal	22/49	1.0 (reference)	
Low SMD + High SATI	14/26	1.54 (.57 - 4.20)	p = 0.395
Both normal	20/49	1.0 (reference)	
Low SMD + High VATI	13/27	0.84 (.30 to 2.38)	p = 0.742
Both normal	21/48	1.0 (reference)	

*All models adjusted for age and stage
